# Efficiency of two nitrification inhibitors (dicyandiamide and 3, 4-dimethypyrazole phosphate) on soil nitrogen transformations and plant productivity: a meta-analysis

**DOI:** 10.1038/srep22075

**Published:** 2016-02-23

**Authors:** Ming Yang, Yunting Fang, Di Sun, Yuanliang Shi

**Affiliations:** 1 Institute of Applied Ecology, Chinese Academy of Sciences, Shenyang 110016, Liaoning, China; 2 University of Chinese Academy of Sciences, Beijing 100049, China; 3 State Key Laboratory of Forest and Soil Ecology, Institute of Applied Ecology, Chinese Academy of Sciences, Shenyang 110164, Liaoning, China

## Abstract

Dicyandiamide (DCD) and 3, 4-dimethypyrazole phosphate (DMPP) are often claimed to be efficient in regulating soil N transformations and influencing plant productivity, but the difference of their performances across field sites is less clear. Here we applied a meta-analysis approach to compare effectiveness of DCD and DMPP across field trials. Our results showed that DCD and DMPP were equally effective in altering soil inorganic N content, dissolve inorganic N (DIN) leaching and nitrous oxide (N_2_O) emissions. DCD was more effective than DMPP on increasing plant productivity. An increase of crop yield by DMPP was generally only observed in alkaline soil. The cost and benefit analysis (CBA) showed that applying fertilizer N with DCD produced additional revenues of $109.49 ha^−1^ yr^−1^ for maize farms, equivalent to 6.02% increase in grain revenues. In comparisons, DMPP application produced less monetary benefit of $15.67 ha^−1^ yr^−1^. Our findings showed that DCD had an advantage of bringing more net monetary benefit over DMPP. But this may be weakened by the higher toxicity of DCD than DMPP especially after continuous DCD application. Alternatively, an option related to net monetary benefit may be achieved through applying DMPP in alkaline soil and reducing the cost of purchasing DMPP products.

Anthropogenic fertilizer N input has now become the main source of new reactive N (Nr) to the global N cycle^1^,^2^. It brings out an increase of almost 50% in food production, which contributes to alleviating global food shortage^3^. However, sub-optimal or over-fertilization have led to an increase of N losses through ammonia (NH_3_) volatilization, nitrate (NO_3_^−^) leaching and nitrous oxide (N_2_O) emissions from soil^4^, which cause severe environmental and ecological problems in water, air and soil^5^. Nitrification inhibitors (NIs) have been developed to mitigate these problems through blocking the first stage of nitrification^6^,^7^.

Among the NIs commercially available, dicyandiamide (DCD) and 3, 4-dimethypyrazole phosphate (DMPP) are the most widely used^8^,^9^. Compared with DMPP, DCD is more widely used in some countries (e.g. New Zealand) as it is cheaper, less volatile and relatively soluble in water^10^. But DMPP has the advantage of lower application rate of one-tenth of DCD dose and minor eco-toxicological side effects for plant growth^11^,^12^,^13^. However, the difference of efficiency at field scale between DCD and DMPP related to altering soil inorganic N, decreasing gaseous emission and increasing plant productivity is less clear, although previous preliminary peer-literatures indicate that DMPP may be more effective lowering NO_3_^−^ leaching and N_2_O emissions than DCD^9^,^14^.

The efficiency of NIs depends on various conditions including soil factors, management factors, crop types, etc. For example, NIs appears to be more effective in soil which has the optimal range of pH values supported for soil nitrification. Meanwhile, efficiency of NIs positively varies with fertilizer N application rates for higher fertilizer N rates input often causing high N loss^9^. N forms may affect the NI’s efficiency through hydrolysis rates to NH_4_^+^-N supplied for soil nitrification. In addition, different crop types showed different responses to the application of NIs, which may be ascribed to their preference to the NH_4_^+^-N and NO_3_^−^-N^1^,^15^. However, previous researchers could not draw general conclusions related to the performances of NIs for the interactions of these effect factors.

Recently, several meta-analyses related to NIs efficiency across sites have been conducted^1^,^9^,^16^,^17^,^18^,^19^,^20^. A comprehensive meta-analysis related to NIs was carry out by Qiao *et al.*^1^, which including soil acidification, N leaching, air pollutant emission, greenhouse gas (GHG) emissions and plant productivity. These researchers also assessed whether the response of those variables will be altered by NI forms, ecosystem types, fertilizer types and soil texture, and performed a cost-benefit analysis (CBA) to quantify the overall impacts of NIs applications by monetary values. However, the efficiency between different nitrification inhibitors (e.g., DCD and DMPP) combined with various conditions as well as the CBA analysis has not been directly compared in these previous meta-analysis studies.

In this study, using a meta-analysis approach, we aimed to compare the efficiency of DCD and DMPP on altering soil inorganic N content, N leaching, gaseous emissions and plant productivity under various conditions including soil pH values, fertilizer N forms, fertilizer N rates and crop types. Additionally, we carried out a CBA for comparing monetary benefits between DCD and DMPP through deducting the application cost from the economic benefit of reducing N’s environmental impacts and increasing plant productivity.

## Results and Discussions

### Soil inorganic N

**Figure 1:**
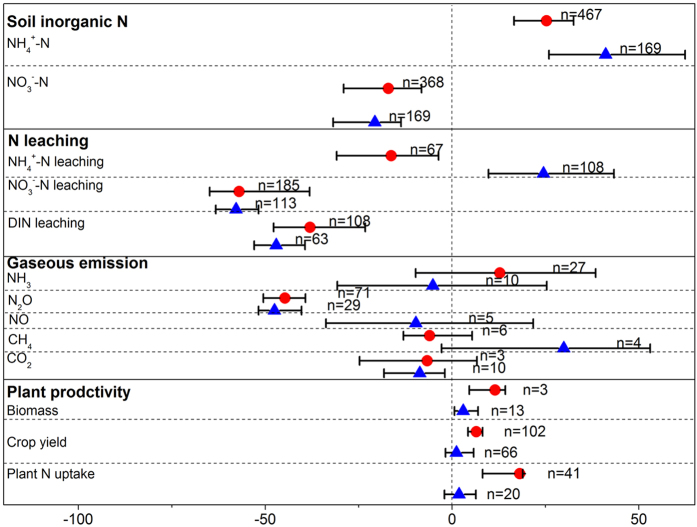
The effect of DCD (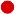) and DMPP (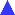) on soil inorganic N, N leaching, gaseous emission and plant productivity as a percentage of the control. Error bars represent 95% confidence intervals (CIs). The effect of fertilizer with NIs was considered significant if the 95% CIs of the effect size did not cover zero. The sample size for each variable is shown next to the point.

We found that DCD and DMPP were equally effective in altering soil inorganic N content at field scale for their confidence intervals overlapping each other (Fig. 1). On average, DCD and DMPP application increased soil NH_4_^+^-N by 25.3% and 41.1%, and decreased soil NO_3_^−^-N content by 17.0% and 20.7%, respectively. These results showed that less than one-tenth of the application rate was enough for DMPP to get a similar inhibition effect compared to DCD^21^.

**Figure 2:**
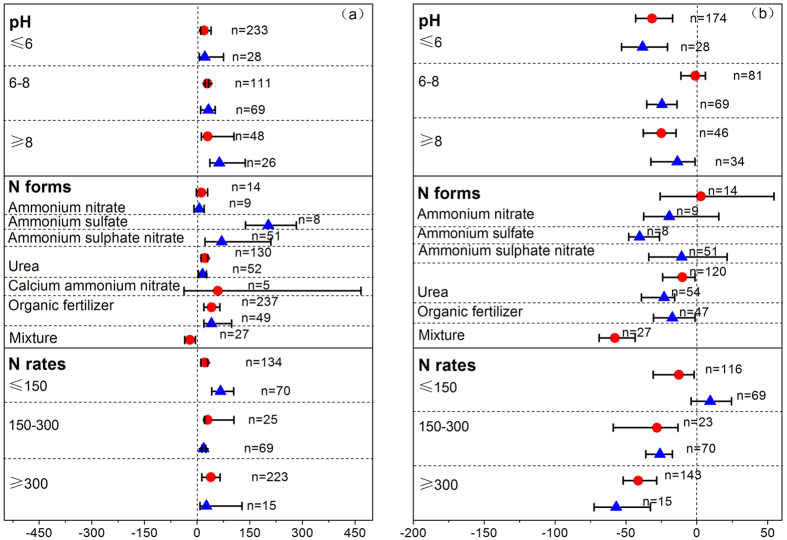
The effect of DCD (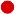) and DMPP (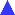) on soil NH_4_^+^-N (**a**) and NO_3_^−^-N content (**b**) as a percentage of the control for different soil pH groups, N forms and N rates. The effect of DCD and DMPP was considered significant if the 95% CI of the effect size did not cover zero. The sample size for each variable is shown next to the point.

By categorizing various factors which may affect the inhibition efficiency, we would acquire the optimal nitrification inhibitor in the specific condition. Their effectiveness were similar for altering soil NH_4_^+^-N and NO_3_^−^-N contents in both acid and alkaline soils (Fig. 2). But the efficiency of DMPP on decreasing soil NO_3_^−^-N content was higher than that of DCD in neutral soils (Fig. 2b).

Many experiments revealed that DCD and DMPP could be used as additive to both chemical and organic fertilizers and their efficiency varied with different N forms^22^,^23^. And this discrepancy may be ascribed to the different rates for hydrolyzing to ionic NH_4_^+^supplied for soil nitrification among various N forms. Both DCD and DMPP were effective in increasing soil NH_4_^+^-N content combined with urea or organic fertilizer (Fig. 2a). DMPP was also effective when combined with inorganic fertilizer (AS or ASN). For soil NO_3_^−^-N content, the optimal fertilizer N forms for DMPP were ammonium sulphate, urea or organic fertilizer (Fig. 2b). But the optimal fertilizer N form for DCD was mixture of animal urine and slurry. For different N application rates, only when fertilizer N application rate was low, DMPP was more effective than DCD for increasing soil NH_4_^+^-N content (Fig. 2a). High fertilizer N input promoted the nitrification inhibition by the two NIs in our study.

### N leaching

The positive effect of NIs on retaining soil NH_4_^+^-N would probably increase the risk of NH_4_^+^-N leaching from soil. But this was not happened in the DCD treatments in our study (Fig. 1). This reduction of NH_4_^+^-N leaching may be caused by the prolonged higher pH values by DCD application which prolonged NH_4_^+^-N retention time and reduced leaching losses^24^ and by the absorption of NH_4_^+^ to clay particles or soil organic matter^25^. Moreover, the greater plant N uptake in the DCD treatments may contribute to this reduction (see Fig. 1, DCD application increased plant N uptake by 18.1%). However, DMPP application significantly increased NH_4_^+^-N leaching which may be related to no significant increase of plant N uptake in DMPP treatments (Fig. 1). The greater amounts of NH_4_^+^-N in leachate treated with DMPP was also observed by other researchers^26^,^27^.

**Figure 3:**
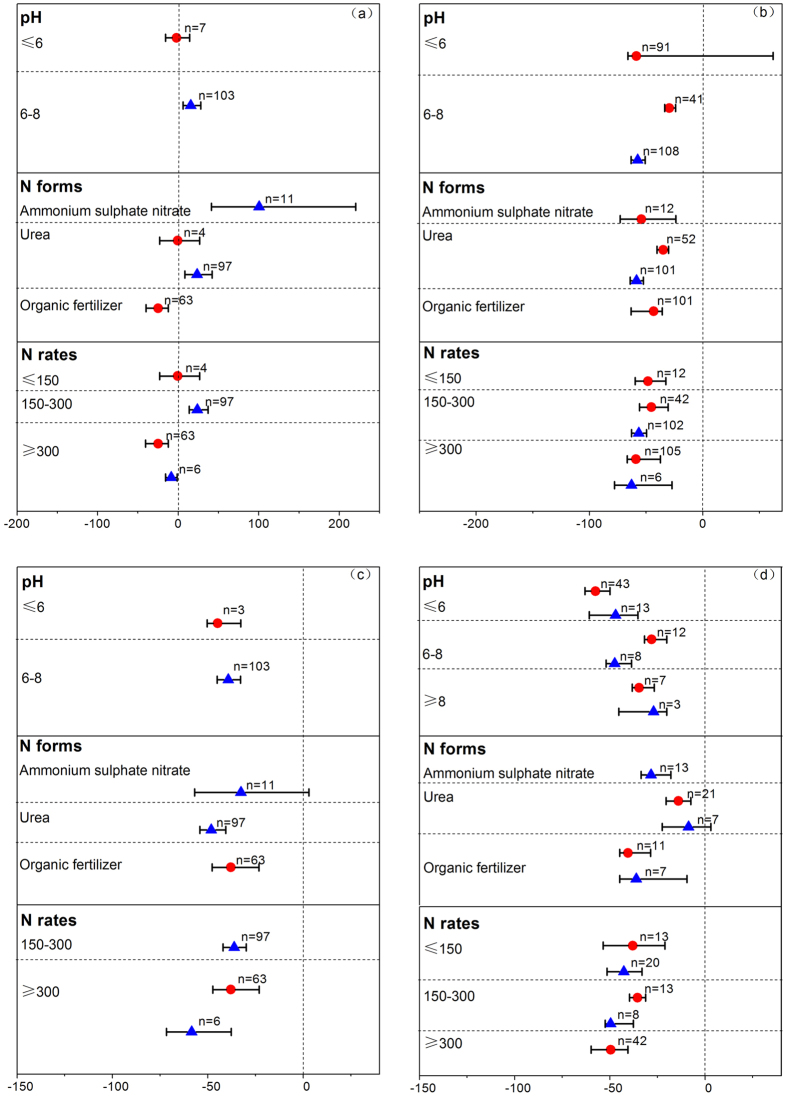
The effect of DCD (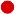) and DMPP (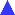) on NH_4_^+^-N leaching (**a**), NO_3_^−^-N leaching (**b**) DIN leaching (**c**) and N_2_O (**d**) emission as a percentage of the control for different soil pH groups, N forms and N rates. The effect of DCD and DMPP was considered significant if the 95% CI of the effect size did not cover zero. The sample size for each variable is shown next to the point.

The effect on N leaching under various conditions was less clear in the previous studies^28^,^29^. The studies related to effect of DCD and DMPP on N leaching were only applied in soil with specific pH value. For example, DCD was applied in acid soils and did not significantly increase NH_4_^+^-N leaching (Fig. 3a). DMPP was also only applied in neutral soil and significantly increased NH_4_^+^-N leaching, which may be ascribed to the increase in NH_4_^+^-N content in neutral soils by DMPP application (results demonstrated in Fig. 2a). But soil NH_4_^+^-N leaching in DCD treatment did not respond to the increase of soil NH_4_^+^-N content by DCD. Among various N forms, DMPP application along with ASN or urea increased soil NH_4_^+^-N leaching which may be caused by an increase in soil NH_4_^+^-N content in addition to the same N form in the fertilizers (Fig. 3a and Fig. 2a). But DCD application significantly decreased soil NH_4_^+^-N leaching under the condition of using organic fertilizers. According to N rates, both NIs were effective in the high fertilizer N rate treatment.

For soil NO_3_^−^-N leaching, both had equal effect on decreasing soil NO_3_^−^-N leaching, as their confidence intervals overlapped with each other (Fig. 1). But in neutral soils or along with urea, DMPP was more effective than DCD (Fig. 3b). Generally, both NIs significantly decreased soil DIN leaching under various conditions except that in the treatment of ASN plus DMPP (Fig. 3c).

### Gaseous emissions

Previous field and laboratory studies on DCD and DMPP applications showed that they could reduce gaseous emission from soil, including NH_3_, N_2_O, NO, CH_4_, CO_2_^23^,^26^,^30^,^31^,^32^,^33^,^34^. There is still a debate on the efficiency of DCD and DMPP on these gaseous emissions at a field scale. For NH_3_ released from soil, our results showed that DCD and DMPP did not alter NH_3_ emissions (Fig. 1). This was consistent with Kim *et al.*^18^ who also observed that no change in NH_3_ loss with DCD application in soil (n = 14).

In our study, DCD and DMPP both significantly decreased soil N_2_O emission by 44.7% and 47.6%, respectively (Fig. 1). And this reduction in N_2_O emission was mainly achieved through reducing NO_3_^−^-N supply for soil denitrification. For DMPP efficiency, Akiyama (2009) estimated that DMPP reduced N_2_O emission by 50% (95% CI: 42% to 55%) through a meta-evaluation with the study number of 12^17^. And we acquired a similar result of 47.6% N_2_O (95% CI: 40.3% to 51.8%) emission decreased by DMPP application (the number of observations was 23) for our meta-analysis including all previous studies (Fig. 1). In addition, we found that both NIs had the equal effectiveness on decreasing soil N_2_O emission. But Akiyama *et al.* (2009) carried out a meta-analysis, and found that DCD was more effective than DMPP in reducing N_2_O emission. This discrepancy may be related to the different numbers of observations between the meta-analysis studies. The number of the observations in our study was larger (n = 71 for DCD; n = 29 for DMPP) than the previous study (n = 42 for DCD; n = 12 for DMPP). And both NIs had similar effectiveness under various conditions except that DMPP was more effective than DCD in neutral soils (Fig. 3d).

For other gaseous emission, only CO_2_ emission was significantly decreased by 8.7% (95% CI: 1.9% to 18.2%) through DMPP application (Fig. 1). This was supported by Weiske *et al.* (2001) who demonstrated that the release of CO_2_ was reduced significantly on average for the 3 years observations. These researchers concluded that DMPP might affect C-mineralization in soil^35^. But when DMPP was applied with ASN or with animal slurry, CO_2_ emission was unaffected^36^. The reasons for discrepancies between the studies remain unclear, calling for more field experiments to confirm.

Methane emission was not significantly altered by DCD and DMPP application (Fig. 1), which potentially limited to the number of observations (n = 6 for DCD; n = 4 for DMPP). But Weiske (2001) found that DMPP apparently stimulated methane oxidation throughout the 3 growing seasons by decreasing 28% in comparison to the control^35^. The mechanism of stimulating oxidation need further study to explain.

### Plant productivity

Our meta-analysis results indicated that DCD significantly increased crop yield by 6.5%, while DMPP did not (increased by 1.2%; 95% CI: −1.6% to 5.8%) (Fig. 1). This was consistent with the results obtained by Abalos *et al.*^9^.

**Figure 4:**
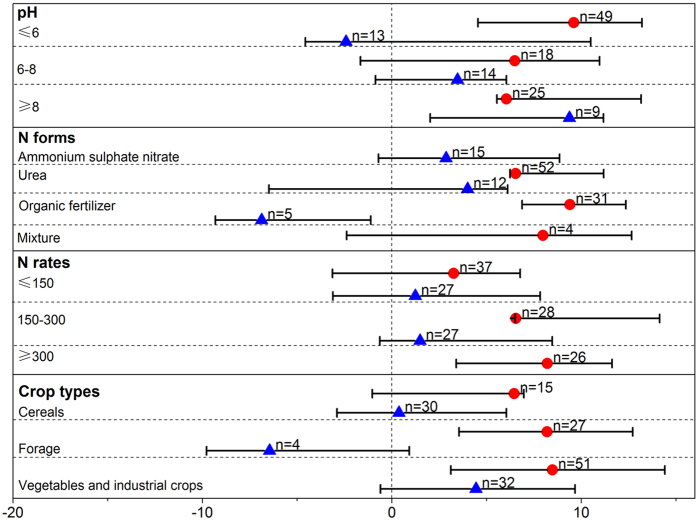
The effect of DCD (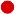) and DMPP (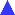) on crop yield as a percentage of the control for different soil pH groups, N forms, N rates, crop types. The effect of DCD and DMPP was considered significant if the 95% CI of the effect size did not cover zero. The sample size for each variable is shown next to the point.

The efficiency of two NIs differed in soil with different pH values (Fig. 4). Higher yields and N uptakes increased by inhibitors (urease and nitrification inhibitors) were associated with higher soil pH values for the rice system^20^. This was not supported by Abalos *et al.* (2014) who found that the overall effect of inhibitors (urease and nitrification inhibitors) on crop yield and NUE for neutral and alkaline soils was decreased through increasing N losses through NH_3_ volatilization. We thus separately evaluated the efficiency of DCD and DMPP in acid, neutral and alkaline soils (Fig. 4). DMPP significantly increased crop yield by 9.4% (95% CI: 2.0% to 11.2%) only in alkaline soil, whereas DCD was both effective in acid and alkaline soil. The most likely reason for the increase of crop yield in alkaline soil was that N loss through soil NH_3_ volatilization was not significant increased by DCD and DMPP application in our study (see Fig. 1).

In terms of N forms, DCD was effective along with organic fertilizer or urea (Fig. 4). But DMPP did not significantly increase crop yield along with various N forms. Moreover, DCD was effective in treatments of medium and high fertilizer N rates. DMPP did not have significant effect on crop yield under different fertilizer N rates treatments.

We also compared the efficiency of two NIs among various crop types (Fig. 4). Both nitrification inhibitors did not significantly alter cereal yield. But forage and vegetables-industrial crop yield were significantly increased by DCD application. This may be attributed to the fact that forage and vegetables-industrial crop generally receive higher N applications than cereals, which may lead to a higher effectiveness of inhibitors. Another potential reason is that cereals are generally harvested for grain rather than aboveground biomass. The response of biomass to DCD or DMPP application in our study verified this reason, in which biomass was more responsive to inhibitor application than crop yield (Fig. 1). Furthermore, DCD significantly increased plant N uptake, but DMPP did not.

### CBA analysis

Compared to N loss factors of conventional N fertilizer practice (*F*_*N*_ = 0.150 for DCD; *F*_*N*_ = 0.156 for DMPP), N loss factors of gaseous emission for DCD and DMPP application were 0.163 and 0.143, respectively (Table 1). N loss factors of DIN leaching for DCD and DMPP application (0.095 for DCD, 0.082 for DMPP) were both lower than that of conventional N fertilizer practice (*F*_*N*_ = 0.154). Overall, DCD and DMPP application resulted in a net reduction of the total N loss by 14.8% and 27.4%, respectively.

The results of CBA case study indicated that a total environmental benefit of $17.61 ha^−1^ and $28.12 ha^−1^ were respectively brought by the application of DCD and DMPP, which was mostly caused by reducing DIN leaching in our study (Table 2). The monetary benefit from DCD application outweighed the cost, leading to an increase in revenue of $109.49 ha^−1^. Based on the mean revenue ($1820 ha^−1^) for a maize farm in US^1^, the revenue increased by DCD application was equivalent to an increase of 6.02% in financial gain. By contrast, DMPP brought less monetary benefit by $15.67 ha^−1^. The revenue produced by DCD application in our study was lower than the revenue ($162.70 ha^−1^) of all NIs estimated by Qiao *et al.* (2015). These results above showed that DCD was more effective than DMPP in total revenue. However, DMPP exclusively focused on alleviating the environmental damage caused by dissolved inorganic N leaching.

## Conclusions

Based on the results from our meta-analysis, we concluded that DCD and DMPP were equally effective in regulating soil N transformations across field sites worldwide. But the performance of DCD in increasing plant productivity was better than that of DMPP. Fertilizer N plus DCD could bring additional revenues of $109.49 ha^−1^ yr^−1^ in maize farms in term of impact of fertilizer N applications plus NIs. DMPP application brought less monetary benefit of $15.67 ha^−1^ yr^−1^ mainly because of no significant effect on crop yield and higher product price. Alternatively, DMPP application in alkaline soil might bring more monetary benefit than DCD even when the price of DMPP products became cheaper. Thus DMPP would be more accepted and popularly applied throughout the world in addition to the lower toxicity for plant growth. These findings highlight the interest in the efficient usage of DCD and DMPP for the future study. The efficiency of DCD and DMPP under continuously application in one given site is needed to examine. Meanwhile, the impact of their toxicity on plant growth and human health is also needed to study after years of application, although the toxicity of both NIs is low. Furthermore, the environmental impact of DCD and DMPP related to the release of NH_3_, NO, CO_2_ and CH_4_ from soil still needs more studies to confirm.

## Methods

### Data collection and selection criteria

Data were acquired by searching existing literature published before June 2015 using the ISI-Web of Science and Google Scholar. The following key words were used for searching such as meta-analysis, efficiency, nitrification inhibitor, DCD, DMPP, inorganic N, N leaching, gaseous emission and plant productivity. And the search terms were complemented with a search through the literature cited in the articles found. Papers were only included if they met the following criteria: 1) only field studies were selected and laboratory incubation studies were excluded; 2) at least one of the selected variables were measured; 3) means and sample sizes had to be reported; 4) treatment replicates were at least of three, etc. Then 81 peer-reviewed publications (49 for DCD, 32 for DMPP) across the world were selected for our analysis (see Supplementary Information).

**Figure 5:**
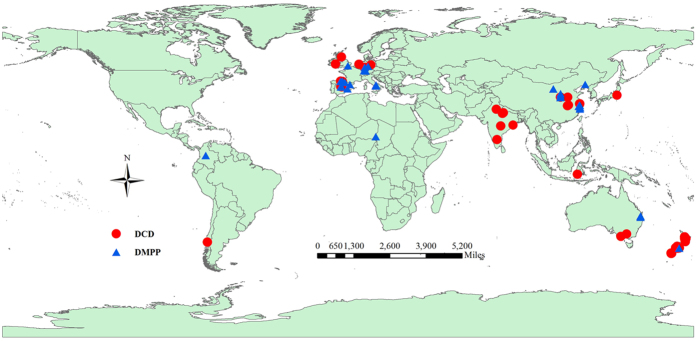
The distribution of study sites around the world for DCD (44 sites)and DMPP (33 sites) application. This figure was generated by ArcGIS software (version 10.1; URL link, http://support.esrichina-bj.cn/2013/0128/1677.html).

For each study, data were collected including study site location (longitude and latitude), soil characteristics (pH values), management measures (fertilizer N types and rates), crop types and the response variables (soil inorganic N content; soil inorganic N leaching; gaseous emission including NH_3_, N_2_O, NO, CO_2_, CH_4_; plant productivity including biomass, crop yield, plant N uptake, etc). For visualizing the distribution of study sites around world, using ArcGIS software (version 10.1; URL link, http://support.esrichina-bj.cn/2013/0128/1677.html), we marked the study sites on the world map through adding data of longitude and latitude for study sites to the map layer. Then the study sites related to DCD application mainly distributed around the world, of which 5 sites located in China, 8 located in Western Europe, 6 located in India, 15 located in New Zealand, respectively. The study sites for DMPP mainly distributed in China (n = 9) and Western Europe (n = 14) (Fig. 5). Data were extracted by Engauge software if the figures were used in the original papers. The standard deviation was either reported or calculated from the standard error and sample size. The number of treatment plots refers to the number of replicate experimental facilities rather than the number of samples per plot. Soil pH was grouped into three types (≤6, 6–8 and ≥8) as used by Linquist *et al.* (2013) and Abalos *et al.* (2014) for comparative purposes^9^,^20^. There were three categories of N fertilizer types: mineral fertilizers including ammonium sulfate (AS), ammonium nitrate (AN), ammonium sulphate nitrate (ASN), calcium ammonium nitrate (CAN), urea, organic fertilizer (animal urine and slurry) and mixture of inorganic and organic fertilizer^9^. Crop type was grouped into three categories including cereals, forage, vegetables-industrial crops^9^.

### Meta-analysis

The mean effect sizes were estimated using the formulas described by Bai *et al.*^37^.


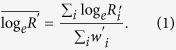


log_e_*R*_*i*_′ and *w*_*i*_′ are log_e_*R*′ and *w*′ of the *i*th observation. log_e_*R*′ is weighed effect size obtained by *w*′ and log_e_*R*.


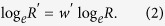


The effect size log_e_*R* was obtained after the log transformation of the ratio of its value in the DCD or DMPP treatment group (*X_t_*) to that in the control group (*X_c_*) for better statistical behavior.


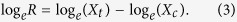


*w*′ was calculated from equation (4) which was adjusted by the total number of observations per site weight, when multiple observations were extracted from the same study. For each study, the weighting factor *w* was calculated as the inverse of the pooled variance (1/*v*).


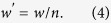


The variance of log_e_*R* was approximated using the following formula:


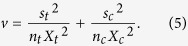


*s*_t_ and *s*_c_ represent the standard deviations of treatment and control groups, respectively; *n*_*t*_ and *n*_*c*_ are the sample sizes for the treatment and control groups, respectively. If no standard deviation in studies was reported, we calculated the average coefficient of variation (CV) within each data set, and then approximated the missing standard deviation by multiplying the reported mean by the average CV^37^.

Then a fixed-effects model option in software Metawin 2.1 was employed for calculation of grouped effect sizes^38^. Confidence intervals (CIs) on the weighted effect size were generated using bootstrapping (9999 iterations). To facilitate explanation, the mean effect size and confidence intervals were transformed back to the percentage change caused by the application of NIs using the following formula:


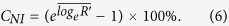


### Cost–benefit analysis

N loss factor (*F*_*N*_) is the ratio of the amount of N fertilizer lost to environment to the amount of N fertilizer applied to soil^1^, which value is acquired from the literatures^39^,^40^,^41^. N loss factor under NI application (*F*_*N* + *NI*_) was estimated by the following formula:


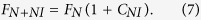


CBA was conducted in US maize farm as a case study. The net monetary benefit of NI application was assessed by summing environmental benefit including DIN leaching, GHG emission, crop yield and cost of purchasing NI products. NI’s impact on the economic value (*M*) of each variable was estimated by the formula.


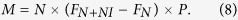


*N* is the mean annual N fertilizer application rate of 125 kg N ha^−1^ yr^−1^ in US cropland^40^. *F*_*N* + *NI*_ and *F*_*N*_ were defined above. *P* is the monetary value related to environmental impacts which is given by previous CBA studies^42^,^43^,^44^.

## Additional Information

**How to cite this article**: Yang, M. *et al.* Efficiency of two nitrification inhibitors (dicyandiamide and 3,4-dimethypyrazole phosphate) on soil nitrogen transformations and plant productivity: a meta-analysis. *Sci. Rep.* **6**, 22075; doi: 10.1038/srep22075 (2016).

## Supplementary Material

Supplementary Information

## Figures and Tables

**Table 1:** N loss factors in the presence (*F*_*N* + *NI*_) and absence (*F*_*N*_) of NIs application.

Nitrogen loss	NIs	*C*_*NI*_	*F*_*N*_	*F*_*N* + *NI*_	Change in N loss (%)
NH_3_ emission	DCD	0.128(n.s)	0.140*	0.158	12.8
	DMPP	−0.051(n.s)	0.140*	0.133	−5.1
N_2_O emission	DCD	−0.447	0.010*	0.006	−44.7
	DMPP	−0.476	0.010*	0.005	−47.6
NO emission	DCD	–	0.006*	–	–
	DMPP	−0.097(n.s)	0.006*	0.005	−9.7
Dissolved inorganic N (DIN) leaching	DCD	−0.380	0.154^†^	0.095	−38.0
	DMPP	−0.471	0.154^†^	0.082	−47.1
Total gaseous N loss^‡^	DCD		0.150	0.163	9.0
	DMPP		0.156	0.143	−8.0
Total N loss^§^	DCD		0.304	0.259	−14.8
	DMPP		0.310	0.225	−27.4

Positive and negative values of *C*_*NI*_ indicated the increase and decrease, respectively, in N loss by DCD or DMPP application. *The source of the data was FAO/IFA^40^. ^†^used by Qiao *et al.*^1^. **n.s** represented no significantly changed by NIs application. –No available data. ^‡^The sum of N loss through NH_3_, N_2_O and NO emission. ^§^The sum of N loss through NH_3_, N_2_O and NO emission and DIN leaching.

**Table 2:** The cost-benefit analysis of NIs application in a maize farm with fertilizer N rate of 125 kgN ha^−1^ yr^−1^.

	Assessed impacts	Cost^1^	Change in N loss under NI (kg N^−1^ha^−1^)*		Monetary response ($ha^−1^)		
			DCD	DMPP	DCD	DMPP	
NH_3_ emission	The cost of human healthdamage	$1.30 kg^−1^N	2.24(n.s)	−0.90(n.s)	−2.91	1.17	
N_2_O emission	The cost of climate change	$1.24 kg^−1^ N	−0.56	−0.59	0.69	0.74	
NO emission	The cost of human healthdamage	$23.00 kg^−1^N	–	−0.07(n.s)	–	1.67	
Dissolved inorganic N leaching	The abatement cost of reducing N from agricultural drainage water	$2.71 kg^−1^ N	−7.32	−9.06	19.82	24.55	
**Sum of the environmental impacts**					**17.61**	**28.12**	
**Variables**	**Assessed impacts**	**Unit price^45^**	**Changes in yield (ton ha^−1^)^#^**		**Monetary response ($ ha^−1^)**		
			**DCD**	**DMPP**	**DCD**	**DMPP**	
Maize production	The benefit of increase in yield	$197.00 ton^−1^	0.60	0.11(n.s)	**118.14**	**21.30**	
**Variables**	**Assessed impacts**	**Unit price^¶^**		**Application rates (kg ha^−1^)^†^**		**Monetary response ($ ha^−1^)**	
		**DCD**	**DMPP**	**DCD**	**DMPP**	**DCD**	**DMPP**
DCD, DMPP	The cost of purchasing DCD or DMPP	$1.75kg^−1^	$27kg^−1^	15.00	1.25	−26.25	−33.75
**Sum of the monetary responses**						**109.49**	**15.67**

For change in N loss under NIs, positive and negative values represent that NIs increases and decrease N losses respectively. For the monetary response, the positive numbers indicate the amount of the economic benefit, whereas the negative ones indicate the amount of the economic cost. ^*^Changes in N loss under NIs = 125 kgN ha^−1^ × (*F*_*N* + *NI*_−*F*_*N*_). *F*_*N*_ and *F*_*N* + *NI*_ values were from Table 1. ^#^The change in maize production = 9.24 ton ha^−1^ × *C*_*NI*_. 9.24 ton ha^−1^ was the mean maize production in US^45^. *C*_*NI*_ was the change in crop yield by NIs application estimated by the current study. ^†^The recommended DCD application rate (15 kg ha^−1^ yr^−1^) was from Di & Cameron^46^. The recommended DMPP application rate (1% N = 1.25 kg ha^−1^) was used by Scheer *et al.*^33^. ^¶^The price of DCD and DMPP were the mean of the market price from the website of Alibaba. **n.s** represented no significantly changed by NIs application. –No available data.
