# Supplementary material for: Efficiency of two nitrification inhibitors (dicyandiamide and 3, 4-dimethypyrazole phosphate) on soil nitrogen transformations and plant productivity: a meta-analysis
Source: Sci Rep. 2016 Feb 23;6:22075. doi: 10.1038/srep22075 (PMC4763264; doi:10.1038/srep22075)
Supplement: Supplementary Information [file srep22075-s1.pdf]

# Efficiency of two nitrification inhibitors (dicyandiamide and 3,4-dimethypyrazole phosphate) on soil nitrogen transformations and plant productivity: a meta-analysis

Ming Yang, Yunting Fang, Di Sun, Yuanliang Shi

List of all the references related to DCD and DMPP used in the meta-analysis.

**References for DCD**

1. Adhya, T. K., Bharati, K., Mohanty, S. R., Ramakrishnan, B., Rao, V. R., & Sethunathan, N., et al. Methane emission from rice fields at cuttack, india. *Nutr. Cycl. Agroecosyst.* **58**, 95-105 (2000).

2. Akiyama, H. *et al.* Nitrification, ammonia-oxidizing communities, and $N_2O$ and $CH_4$ fluxes in an imperfectly drained agricultural field fertilized with coated urea with and without dicyandiamide. *Biol. Fertil. Soils.* **49**, 213-223 (2012).

3. Ball, B. C., Cameron, K. C., Di, H. J. & Moore, S. Effects of trampling of a wet dairy pasture soil on soil porosity and on mitigation of nitrous oxide emissions by a nitrification inhibitor, dicyandiamide. *Soil Use Manage.* **28**, 194-201 (2012).

4. Banerjee, B., Pathak, H., & Aggarwal, P. Effects of dicyandiamide, farmyard manure and irrigation on crop yields and ammonia volatilization from an alluvial soil under a rice (*Oryza sativa* L.)-wheat (*Triticum aestivum* L.) cropping system. *Biol. Fertil. Soils.* **36**, 207-214 (2002).

5. Cui, M. *et al.* Effective mitigation of nitrate leaching and nitrous oxide emissions in intensive vegetable production systems using a nitrification inhibitor, dicyandiamide. *J. Soils Sediments.* **11**, 722-730 (2011).

6. Dai, Y., Di, H. J., Cameron, K. C. & He, J. Z. Effects of nitrogen application rate and a nitrification inhibitor dicyandiamide on methanotroph abundance and methane uptake in a grazed pasture soil. *Environ. Sci. Pollut. Res.*. **20**, 8680-8689 (2013).

7. Di, H. J. & Cameron, K. C. How does the application of different nitrification inhibitors affect nitrous oxide emissions and nitrate leaching from cow urine in grazed pastures. *Soil Use Manage.* **28**, 54-61 (2012).

8. Di, H. J. & Cameron, K. C. Mitigation of nitrous oxide emissions in spray-irrigated grazed grassland by treating the soil with dicyandiamide, a nitrification inhibitor. *Soil Use Manage.* **19**, 284-290 (2003).

9. Di, H. J. & Cameron, K. C. Nitrous oxide emissions from two dairy pasture soils as affected by different rates of a fine particle suspension nitrification inhibitor, dicyandiamide. *Biol. Fertil. Soils.* **42**, 472-480 (2006).

10. Di, H. J. & Cameron, K. C. Reducing environmental impacts of agriculture by using a fine particle suspension nitrification inhibitor to decrease nitrate leaching from grazed pastures. *Agric. Ecosyst. Environ.* **109**, 202-212 (2005).

11. Di, H. J. & Cameron, K. C. Treating grazed pasture soil with a nitrification inhibitor, eco-n (TM), to decrease nitrate leaching in a deep sandy soil under spray irrigation - a lysimeter study. *N. Z. J. Agric. Res.* **47**, 351-361 (2004).

12. Di, H. J., Cameron, K. C. & Sherlock, R. R. Comparison of the effectiveness of a nitrification inhibitor, dicyandiamide, in reducing nitrous oxide emissions in four different soils under different climatic and management conditions. *Soil Use Manage.* **23**, 1-9 (2007).

13. Di, H. J. *et al.* Nitrous oxide emissions from grazed grassland as affected by a nitrification inhibitor, dicyandiamide, and relationships with ammonia-oxidizing bacteria and archaea. *J. Soils Sediments.* **10**, 943-954 (2010).

14. Ding, W., Yu, H. & Cai, Z. Impact of urease and nitrification inhibitors on nitrous oxide emissions from fluvo-aquic soil in the North China Plain. *Biol. Fertil. Soils.* **47**, 91-99 (2011).

15. Giltrap, D. L., Singh, J., Saggar, S. & Zaman, M. A preliminary study to model the effects of a nitrification inhibitor on nitrous oxide emissions from urine-amended pasture. *Agric. Ecosyst. Environ.* **136**, 310-317 (2010).

16. Harris, R. *et al.* Can nitrogen fertiliser and nitrification inhibitor management influence $N_2O$ losses from high rainfall cropping systems in South Eastern Australia. *Nutr. Cycl. Agroecosyst.* **95**, 269-285 (2013).

17. Irigoyen, I., Lamsfus, C., Aparicio-Tejo, P. & Muro, J. The influence of 3,4-dimethylpyrazole phosphate and dicyandiamide on reducing nitrate accumulation in spinach under Mediterranean conditions. *J. Agric. Sci.* **144**, 555-562 (2006).

18. Jumadi, O. *et al.* Influences of chemical fertilizers and a nitrification inhibitor

on greenhouse gas fluxes in a corn (*Zea mays* L.) field in Indonesia. *Microbes Environ.* **23**, 29-34 (2008).

19. Kelly, K. B., Phillips, F. A. & Baigent, R. Impact of dicyandiamide application on nitrous oxide emissions from urine patches in northern Victoria, Australia. *Aust. J. Exp. Agr.* **48**, 156-159(2008).

20. Kiran, U. & Patra, D. D. Influence of natural essential oils and their by-products as nitrification retarders in regulating nitrogen utilization for Japanese mint in sandy loam soils of subtropical central India. *Agric. Ecosyst. Environ.* **94**, 237-245(2003).

21. Klein, C. A. M. D. *et al.* Repeated annual use of the nitrification inhibitor dicyandiamide (DCD) does not alter its effectiveness in reducing $N_2O$ emissions from cow urine. *Anim. Feed Sci. Technol.* **166 / 167**, 480-491 (2011).

22. Klein, C. A. M. D., Logtestijn, R. S. P. V., Meer, H. G. V. D. & Geurink, J. H. Nitrogen losses due to denitrification from cattle slurry injected into grassland soil with and without a nitrification inhibitor. *Plant Soil.* **183**, 161-170 (1996).

23. Kumar, U., Jain, M. C., Pathak, H., Kumar, S. & Majumdar, D. Nitrous oxide emission from different fertilizers and its mitigation by nitrification inhibitors in irrigated rice. *Biol. Fertil. Soils.* **32**, 474-478(2000).

24. Liu, C., Wang, K. & Zheng, X. Effects of nitrification inhibitors (DCD and DMPP) on nitrous oxide emission, crop yield and nitrogen uptake in a wheat–maize cropping system. *Biogeosciences.* **10**, 2427-2437 (2013).

25. Ma, Y. *et al.* Mitigation of nitrous oxide emissions from paddy soil under conventional and no-till practices using nitrification inhibitors during the winter wheat-growing season. *Biol. Fertil. Soils.* **49**, 627-635 (2013).

26. Macadam, X. M. B. *et al.* Dicyandiamide and 3,4-dimethylpyrazole phosphate decrease $N_2O$ emissions from grassland but dicyandiamide produces deleterious effects in clover. *J. Plant Physiol.* **160**, 1517-1523 (2003).

27. Majumdar, D., Kumar, S., Pathak, H., Jain, M. C. & Kumar, U. Reducing nitrous oxide emission from an irrigated rice field of North India with nitrification inhibitors. *Agric. Ecosyst. Environ.* **81**, 163-169 (2000).

28. Majumdar, D., Pathak, H., Kumar, S. & Jain, M. C. Nitrous oxide emission from a sandy loam Inceptisol under irrigated wheat in India as influenced by different nitrification inhibitors. *Agric. Ecosyst. Environ.* **91**, 283-293 (2002).

29 Malla, G. *et al.* Mitigating nitrous oxide and methane emissions from soil in rice-wheat system of the Indo-Gangetic plain with nitrification and urease inhibitors. *Chemosphere.* **58**, 141-147 (2005).

30 McTaggart, I. P., Clayton, H., Parker, J., Swan, L. & Smith, K. A. Nitrous oxide emissions from grassland and spring barley, following N fertiliser application with and without nitrification inhibitors. *Biol. Fertil. Soils.* **25**, 261-268(1997).

31 Merino, P., Estavillo, J. M., Besga, G., Pinto, M. & Gonzalez-Murua, C. Nitrification and denitrification derived $N_2O$ production from a grassland soil under application of DCD and Actilith $F_2$. *Nutr. Cycl. Agroecosyst.* **60**, 9-14

(2001).

32 Merino, P., Menendez, S., Pinto, M., Gonzalez-Murua, C. & Estavillo, J. M. 3,4-dimethylpyrazole phosphate reduces nitrous oxide emissions from grassland after slurry application. *Soil Use Manage.* **21**, 53-57 (2005).

33 Moir, J. L., Cameron, K. C. & Di, H. J. Effects of the nitrification inhibitor dicyandiamide on soil mineral N, pasture yield, nutrient uptake and pasture quality in a grazed pasture system. *Soil Use Manage.* **23**, 111-120 (2007).

34 Monaghan, R. M., Smith, L. C. & Klein, C. A. M. D. The effectiveness of the nitrification inhibitor dicyandiamide (DCD) in reducing nitrate leaching and nitrous oxide emissions from a grazed winter forage crop in southern New Zealand. *Agric. Ecosyst. Environ.* **175**, 29-38 (2013).

35 Monaghan, R. M., Smith, L. C. & Ledgard, S. F. The effectiveness of a granular formulation of dicyandiamide (DCD) in limiting nitrate leaching from a grazed dairy pasture. *N. Z. J. Agric. Res.* **52**, 145-159 (2009).

36 O'Connor, P. J., Lynch, M. B., Cahalan, E., O'Donovan, M. & Hennessy, D. The effect of the nitrification inhibitor dicyandiamide (DCD) on spring and annual herbage production in urine patches when applied in late summer or early autumn. *Grass Forage Sci.* **68**, 564-576 (2013).

37 Patra, D. D. *et al.* Nimin and Mentha spicata oil as nitrification inhibitors for optimum yield of Japanese mint. *Commun. Soil Sci. Plant Anal.* **33**, 451-460 (2002).

38 Qiu, W., Di, H. J., Cameron, K. C. & Hu, C. Nitrous oxide emissions from

animal urine as affected by season and a nitrification inhibitor dicyandiamide. *J. Soils Sediments.* **10**, 1229-1235 (2010).

39 Ram, M., Patra, D. D. & Singh, D. V. Effect of nitrification inhibitors on herb and essential oil yield of Japanese mint on sandy soil. *Fertilizer Research.* **44**, 17-21 (1996).

40 Rao, E. & Puttanna, K. Nitrification and ammonia volatilization losses from urea and dicyandiamide-treated urea in a sandy loam soil. *Plant Soil.* **97**, 201-206 (1987).

41 Smith, L. C., Monaghan, R. M., Ledgard, S. F. & Catto, W. D. The effectiveness of different nitrification inhibitor formulations in limiting nitrate accumulation in a Southland pastoral soil. *N. Z. J. Agric. Res.* **48**, 517-529 (2005).

42 Vallejo, A., García-Torres, L., Díez, J. A., Arce, A. & López-Fernández, S. Comparison of N losses ($NO_3^-$, $N_2O$, NO) from surface applied, injected or amended (DCD) pig slurry of an irrigated soil in a Mediterranean climate. *Plant Soil.* **272**, 313-325 (2005).

43 Vistoso, E., Alfaro, M., Saggar, S. & Salazar, F. Effect of nitrogen inhibitors on nitrous oxide emissions and pasture growth after an autumn application in volcanic soil. *Chil. J. Agric. Res.* **72**, 133-139 (2012).

44 Wadman, W. P., Neeteson, J. J. & Wijnen, G. in *Nitrogen in Organic Wastes* (eds Hansen, J. A. & Henriksen, K. A. J.) 304-314 (Academic Press, 1989).

45 Watkins, N. L., Schipper, L. A., Sparling, G. P., Thorrold, B. & Balks, M.

Multiple small monthly doses of dicyandiamide (DCD) did not reduce denitrification in Waikato dairy pasture. *N. Z. J. Agric. Res.* **56**, 37-48 (2013).

46 Welten, B. G., Ledgard, S. F., Schipper, L. A. & Judge, A. A. Effect of amending cattle urine with dicyandiamide on soil nitrogen dynamics and leaching of urinary-nitrogen. *Agric. Ecosyst. Environ.* **167**, 12-22 (2013).

47 Zaman, M. & Blennerhassett, J. D. Effects of the different rates of urease and nitrification inhibitors on gaseous emissions of ammonia and nitrous oxide, nitrate leaching and pasture production from urine patches in an intensive grazed pasture system. *Agric. Ecosyst. Environ.***136**, 236-246 (2010).

48. Zaman, M. & Nguyen, M. L. How application timings of urease and nitrification inhibitors affect N losses from urine patches in pastoral system. *Agric. Ecosyst. Environ.* **156**, 37-48 (2012).

49. Zaman, M., Saggar, S., Blennerhassett, J. D. & Singh, J. Effect of urease and nitrification inhibitors on N transformation, gaseous emissions of ammonia and nitrous oxide, pasture yield and N uptake in grazed pasture system. *Soil Biol. Biochem.* **41**, 1270-1280 (2009).

**References for DMPP**

1. Bañuls, J., Quiñones, A., Primo-Millo, E. & Legaz, F. A new nitrification inhibitor (DMPP) improves the nitrogen fertilizer efficiency in citrus-growing systems. in *Plant Nutrition: Developments in Plant and Soil Sciences*, Vol. 92 (eds Horst, W. J. *et al.*), 762-763 (Springer Netherlands, 2001).
2. Díez López, J. A., Hernáiz, P. J., Arauzo, M. & Carrasco Martín, I. Effect of a nitrification inhibitor (DMPP) on nitrate leaching and maize yield during two growing seasons. *Span. J. Agric. Res.* **6**, 294-303 (2008).
3. De Antoni Migliorati, M. *et al.* Influence of different nitrogen rates and DMPP nitrification inhibitor on annual $N_2O$ emissions from a subtropical wheat–maize cropping system. *Agric. Ecosyst. Environ.* **186**, 33-43 (2014).
4. Di, H. J. & Cameron, K. C. How does the application of different nitrification inhibitors affect nitrous oxide emissions and nitrate leaching from cow urine in grazed pastures. *Soil Use Manage.* **28**, 54-61 (2012).
5. Dittert, K., Bol, R., King, R., Chadwick, D. & Hatch, D. Use of a novel nitrification inhibitor to reduce nitrous oxide emission from $^{15}$N-labelled dairy slurry injected into soil. *Rapid Commun. Mass Spectrom.* **15**, 1291-1296 (2001).
6. Du A, G., Ju, X. T., Zhang, F. S., Chen, L. & Xie, L. S. Transformations of new fertilizer Entec26 in soil and it's effect on maize yield. *J. Agric. Sci. Technol.* 47-51 (2003).
7. García-Castro, A. & Restrepo-Díaz, H. Evaluation of fertilization with uncoated urea and 3,4-dimethylpyrazole phosphate (DMPP)-coated urea on

nitrogen leaching and rose (*Rosa* spp.) yield. *Chil. J. Agric. Res.* **73**, 20-21 (2013).

8. Garcia-Castro, A., Restrepo-Diaz, H. & Florez-Roncancio, V. J. The use of the nitrification inhibitor 3, 4 dimethylpyrazol phosphate (DMPP) on the growth of rose plants cultivated in soil and coconut fibre. *International Symposium on Growing Media, Composting and Substrate Analysis.* **1013**, 285-290 (2013).
9. Irigoyen, I., Lamsfus, C., Aparicio-Tejo, P. & Muro, J. The influence of 3,4-dimethylpyrazole phosphate and dicyandiamide on reducing nitrate accumulation in spinach under Mediterranean conditions. *J. Agric. Sci.* **144**, 555-562 (2006).
10. Li, H., Chen, Y. X., Liang, X. Q., Lian, Y. F. & Li, W. H. Mineral-nitrogen leaching and ammonia volatilization from a rice-rapeseed system as affected by 3,4-dimethylpyrazole phosphate. *J. Environ. Qual.* **38**, 2131-2137 (2009).
11. Li, H. *et al.* Effect of nitrification inhibitor DMPP on nitrogen leaching, nitrifying organisms, and enzyme activities in a rice-oilseed rape cropping system. *J. Environ. Sci.* **20**, 149-155 (2008).
12. Linzmeier, W., Gutser, R., & Schmidhalter, U. Nitrous oxide emission from soil and from a nitrogen-15-labelled fertilizer with the new nitrification inhibitor 3,4-dimethylpyrazole phosphate (DMPP). *Biol. Fertil. Soils.* **34**, 103-108 (2001).
13. Liu, C., Wang, K. & Zheng, X. Effects of nitrification inhibitors (DCD and DMPP) on nitrous oxide emission, crop yield and nitrogen uptake in a

wheat–maize cropping system. *Biogeosciences.* **10**, 2427-2437 (2013).

14. Macadam, X. M. B. *et al.* Dicyandiamide and 3,4-dimethylpyrazole phosphate decrease $N_2O$ emissions from grassland but dicyandiamide produces deleterious effects in clover. *J. Plant Physiol.* **160**, 1517-1523 (2003).

15. Menéndez, S., Barrena, I., Setien, I., González-Murua, C. & Estavillo, J. M. Efficiency of nitrification inhibitor DMPP to reduce nitrous oxide emissions under different temperature and moisture conditions. *Soil Biol. Biochem.* **53**, 82-89, (2012).

16. Menéndez, S., Merino, P., Pinto, M., González-Murua, C. & Estavillo, J. 3, 4-Dimethylpyrazol phosphate effect on nitrous oxide, nitric oxide, ammonia, and carbon dioxide emissions from grasslands. *J. Environ. Qual.* **35**, 973-981 (2006).

17. Menéndez, S., Merino, P., Pinto, M., González-Murua, C. & Estavillo, J. Effect of N-(-butyl) thiophosphoric triamide and 3,4 dimethylpyrazole phosphate on gaseous emissions from grasslands under different soil water contents. *J. Environ. Qual.* **38**, 27-35 (2009).

18. Merino, P., Menendez, S., Pinto, M., Gonzalez-Murua, C. & Estavillo, J. M. 3,4-dimethylpyrazole phosphate reduces nitrous oxide emissions from grassland after slurry application. *Soil Use Manage.* **21**, 53-57 (2005).

19. Pasda, G. et al. The new nitrification inhibitor DMPP (ENTEC®) - effects on yield and quality of agricultural and horticultural crops. in *Plant nutrition: Developments in Plant and Soil Sciences*, Vol. 92 (eds Horst, W. J. *et al.*),

766-767 (Springer Netherlands, 2001).

20. Pasda, G., Hähndel, R., & Zerulla, W. Effect of fertilizers with the new nitrification inhibitor DMPP (3,4-dimethylpyrazole phosphate) on yield and quality of agricultural and horticultural crops. *Biol. Fertil. Soils.* **34**, 85-97 (2001).

21. Pfab, H. *et al*. Influence of a nitrification inhibitor and of placed N-fertilization on $N_2O$ fluxes from a vegetable cropped loamy soil. *Agric. Ecosyst. Environ*. **150**, 91-101 (2012).

22. Scheer, C. *et al.* Impact of nitrification inhibitor (DMPP) on soil nitrous oxide emissions from an intensive broccoli production system in sub-tropical Australia. *Soil Biol. Biochem.* **77**, 243-251 (2014).

23. Serna, M. D., Banuls, J., Quinones, A., Primo-Millo, E. & Legaz, F. Evaluation of 3,4-dimethylpyrazole phosphate as a nitrification inhibitor in a Citrus-cultivated soil. *Biol. Fertil. Soils.* **32**, 41-46 (2000).

24. Villar, J. M. & Guillaumes, E. Use of nitrification inhibitor DMPP to improve nitrogen recovery in irrigated wheat on a calcareous soil. *Span. J. Agric. Res.* **8**, 1218-1230 (2010).

25. Vitale, L. *et al.* Effects of 3,4-dimethylphyrazole phosphate-added nitrogen fertilizers on crop growth and $N_2O$ emissions in Southern Italy. *Plant Soil Environ.* **59**, 517-523 (2013).

26. Weiske, A., Benckiser, G., Herbert, T. & Ottow, J. Influence of the nitrification inhibitor 3,4-dimethylpyrazole phosphate (DMPP) in comparison to

dicyandiamide (DCD) on nitrous oxide emissions, carbon dioxide fluxes and methane oxidation during 3 years of repeated application in field experiments. *Biol. Fertil. Soils.* **34**, 109-117 (2001).

27. Weiske, A., Benckiser, G. & Ottow, J. C. Effect of the new nitrification inhibitor DMPP in comparison to DCD on nitrous oxide ($N_2O$) emissions and methane ($CH_4$) oxidation during 3 years of repeated applications in field experiments. *Nutr. Cycl. Agroecosyst.* **60**, 57-64 (2001).

28. Wu, S. f. *et al.* Effects of a new nitrification inhibitor 3,4-dimethylpyrazole phosphate (DMPP) on nitrate and potassium leaching in two soils. *J. Environ. Sci.* **19**, 841-847 (2007).

29. Wu, S. F., Wu, L. H., Yin, Y. M., Yang, S. D. & Li, Q. Effects of compound fertilizer with nitrification inhibitor on the yield and nutritional quality of water melon andcucumber. *Journal. Agro-Environ. Sci.* 1432-1435 (2006).

30. Wu, S. F., Wu, L. H., Yin, Y. M., Yang, S. D. & Chen, X. Y. Effects of DMPP-compound fertilizer on greenhouse celery growth and nutritional quality. *Chinese Journal of Applied Ecology*. 383-388 (2007).

31. Yu, Q., Ye, X., Chen, Y., Zhang, Z. & Tian, G. Influences of nitrification inhibitor 3,4-dimethyl pyrazole phosphate on nitrogen and soil salt-ion leaching. *J. Environ. Sci.* **20**, 304-308 (2008).

32. Zhang, L. *et al.* Fate of applied urea $^{15}N$ in a soil-maize system as affected by urease inhibitor and nitrification inhibitor. *Plant Soil Environ.* **56**, 8-15 (2010).
